# Nutrient-mediated tree growth responses are governed by internal resource allocation: intra-organ water–carbon trade-offs and inter-organ carbon concentration gradient

**DOI:** 10.1093/treephys/tpag109

**Published:** 2026-08-08

**Authors:** Hui Shao, Huimin Wang, Frederick C Meinzer, Xiaoqin Dai, Shengwang Meng, Liang Kou, Decai Gao, Fusheng Chen, Xiaoli Fu

**Affiliations:** Qianyanzhou Ecological Research Station, Key Laboratory of Ecosystem Network Observation and Modeling, Institute of Geographic Sciences and Natural Resources Research, Chinese Academy of Sciences, 11A Datun Road, Chaoyang District, Beijing 100101, China; College of Resources and Environment, University of Chinese Academy of Sciences, No. 1 Yanqihu East Road, Huairou District, Beijing 101408, China; Qianyanzhou Ecological Research Station, Key Laboratory of Ecosystem Network Observation and Modeling, Institute of Geographic Sciences and Natural Resources Research, Chinese Academy of Sciences, 11A Datun Road, Chaoyang District, Beijing 100101, China; College of Resources and Environment, University of Chinese Academy of Sciences, No. 1 Yanqihu East Road, Huairou District, Beijing 101408, China; Department of Forest Ecosystems and Society, Oregon State University, 1500 SW Jefferson Way, Corvallis, OR 97331, USA; Qianyanzhou Ecological Research Station, Key Laboratory of Ecosystem Network Observation and Modeling, Institute of Geographic Sciences and Natural Resources Research, Chinese Academy of Sciences, 11A Datun Road, Chaoyang District, Beijing 100101, China; College of Resources and Environment, University of Chinese Academy of Sciences, No. 1 Yanqihu East Road, Huairou District, Beijing 101408, China; Qianyanzhou Ecological Research Station, Key Laboratory of Ecosystem Network Observation and Modeling, Institute of Geographic Sciences and Natural Resources Research, Chinese Academy of Sciences, 11A Datun Road, Chaoyang District, Beijing 100101, China; College of Resources and Environment, University of Chinese Academy of Sciences, No. 1 Yanqihu East Road, Huairou District, Beijing 101408, China; Qianyanzhou Ecological Research Station, Key Laboratory of Ecosystem Network Observation and Modeling, Institute of Geographic Sciences and Natural Resources Research, Chinese Academy of Sciences, 11A Datun Road, Chaoyang District, Beijing 100101, China; College of Resources and Environment, University of Chinese Academy of Sciences, No. 1 Yanqihu East Road, Huairou District, Beijing 101408, China; Qianyanzhou Ecological Research Station, Key Laboratory of Ecosystem Network Observation and Modeling, Institute of Geographic Sciences and Natural Resources Research, Chinese Academy of Sciences, 11A Datun Road, Chaoyang District, Beijing 100101, China; College of Resources and Environment, University of Chinese Academy of Sciences, No. 1 Yanqihu East Road, Huairou District, Beijing 101408, China; Jiangxi Provincial Key Laboratory of Silviculture, College of Forestry, Jiangxi Agricultural University, No. 1101 Zhimin Road, Economic & Technological Development Zone, Nanchang, Jiangxi 330045, China; Qianyanzhou Ecological Research Station, Key Laboratory of Ecosystem Network Observation and Modeling, Institute of Geographic Sciences and Natural Resources Research, Chinese Academy of Sciences, 11A Datun Road, Chaoyang District, Beijing 100101, China; College of Resources and Environment, University of Chinese Academy of Sciences, No. 1 Yanqihu East Road, Huairou District, Beijing 101408, China; Jiangxi Provincial Key Laboratory of Silviculture, College of Forestry, Jiangxi Agricultural University, No. 1101 Zhimin Road, Economic & Technological Development Zone, Nanchang, Jiangxi 330045, China

**Keywords:** growth rate, nonstructural carbohydrate, nutrient gradient, water storage, water–carbon interdependency

## Abstract

Nutrient deposition is reshaping forest productivity, yet the physiological mechanisms linking nitrogen (N) and phosphorus (P) enhancement to tree growth remain incompletely resolved. We propose that internal resource allocation strategies—specifically intra-organ trade-offs between water and carbohydrate resources and inter-organ carbohydrate concentration gradient—mediate nutrient-driven tree growth responses. Using a long-term fertilization experiment in subtropical *Cunninghamia lanceolata* (Lamb.) Hook. plantations, we measured relative water content (RWC) and traditional resource concentrations [soluble sugar (SS) concentration; starch (ST) concentration] during drought season. We rank-transformed RWC, SS and ST within each organ type across all samples. Intra-organ water–carbon trade-offs were quantified as two normalized ratios: *ln*(*RWC*_rank_/*SS*_rank_) for RWC:SS and *ln*(*RWC*_rank_/*ST*_rank_) for RWC:ST, where higher values indicate greater solute dependence for turgor maintenance or greater ST storage volume fraction, respectively. The inter-organ carbon concentration gradient was quantified as differences in SS or ST between leaves and twigs (*SS_leaf-twig_*, *ST_leaf-twig_*); and between absorptive roots and transport roots*.* We demonstrated three key nutrient-specific responses: (i) N addition promoted transport root *ln*(*RWC*_rank_/*SS*_rank_) while enhancing leaf *ln*(*RWC*_rank_/*ST*_rank_), amplifying *ST_leaf-twig_*; (ii) P addition reduced leaf and twig *ln*(*RWC*_rank_/*SS*_rank_) and (iii) combined N+P addition reduced twig *ln*(*RWC*_rank_/*SS*_rank_), while increasing leaf and twig *ln*(*RWC*_rank_/*ST*_rank_). These reorganization patterns had direct growth consequences: while traditional resource concentrations explained 26.0% of growth variation, incorporating derived attributes increased explanatory power by 43.5% (to 37.3% total variance explained). Notably, the twig *ln*(*RWC*_rank_/*ST*_rank_) emerged as the single strongest growth predictor, where N+P induced ST dominance correlated with enhanced growth rate. Nitrogen enrichment enhanced SS dominance in transport roots, a pattern that may occur at the expense of stem growth. Our results establish an internal resource allocation framework that mechanistically links nutrient-mediated carbon management patterns to forest productivity under global change, revealing how N+P co-enrichment synergistically optimizes carbon resource storage and utilization beyond single-nutrient effects.

## Introduction

Forests represent the largest terrestrial carbon sink, with their carbon storage dynamics playing a pivotal role in the global biogeochemical cycle (Liu et al. 2025). Recent decades have witnessed dramatic alterations in nutrient regimes, particularly through atmospheric nitrogen (N) and phosphorus (P) deposition (Tian et al. 2024, Zhu et al. 2025). While both elements have seen elevated deposition rates, the magnitude of N deposition far exceeds that of P and has increased more rapidly in tropical and subtropical regions (Du et al. 2016, Pan et al. 2021, Tian et al. 2024), pushing forests in these regions toward N saturation (Cen et al. 2025). Understanding the physiological mechanisms underlying tree growth responses to these nutrient perturbations is essential for predicting forest ecosystem trajectories under global change.

Nonstructural carbohydrates (NSCs)—comprising soluble sugar (SS) and starch (ST)—serve as central hubs of tree metabolism, fueling growth and stress responses (Hartmann and Trumbore 2016, Pratt and Jacobsen 2017, Pratt et al. 2021). Soluble sugar acts as a readily accessible metabolic currency, providing both immediate substrates and osmoprotection, while ST serves as a long-term carbon reserve that buffers temporal mismatches between photosynthetic carbon supply and physiological consumption demand during the drought (Li and McDowell 2026). Environmental stress alters carbon allocation between organs, with direct consequences for growth (Cabon 2026). For example, under well-watered conditions, reduced carbon flow from the canopy to the roots favors stem growth, whereas under drought, preferential carbon allocation to the roots limits stem growth (Hartmann et al. 2020, Reinelt et al. 2023, Cabon 2026). Shifts in water relations are also central to how trees adapt to drought (McDowell et al. 2022). Relative water content (RWC) directly measures the dehydration state of plant organs and integrates the trade-offs among water supply, loss and retention during drought (Martinez-Vilalta et al. 2019). Comparisons of RWC across organs provide a window into the plant’s internal sensing of water deficits, with Duan et al. (2023) demonstrating that root RWC declined more rapidly than that of leaf and stem during drought, highlighting its potential as a warning signal of hydraulic failure. Emerging paradigms emphasize tight coupling between NSC dynamics and hydraulic processes: drought-induced xylem embolism restricts phloem water flux, increasing phloem sap viscosity in petioles (Epron et al. 2019, Jin et al. 2019, Peters et al. 2020) and impairing SS translocation to twigs (Chapotin et al. 2006, Preisler et al. 2022, Duan et al. 2023, Piper and Fajardo 2024). This phloem limitation constrains NSC availability for stem growth, while drought concurrently promotes root SS accumulation to enhance osmotic adjustment and water uptake (Huang et al. 2023, Voothuluru et al. 2024)—critical for maintaining tissue hydration status (e.g. RWC).

Nutrient enrichment fundamentally regulates plant water–carbon interactions and inter-organ carbon concentration patterns. For example, N enrichment typically induces xylem conduit enlargement (wider tracheids/vessels), augmenting hydraulic conductivity at the cost of increased drought-induced embolism (Wang et al. 2017, Zhang et al. 2018, Y.Lu et al. 2024). Fine roots—the most embolism-prone organs (Jiang et al. 2022)—exhibit compensatory regeneration following stress-relief. While new root growth enhances water uptake efficiency, it incurs substantial carbon costs (Brunner et al. 2015). This metabolic trade-off likely underpins the observed accumulation of NSC in roots under N addition (Huang et al. 2021*b*, Schönbeck et al. 2021, Wang et al. 2021). Additionally, N enrichment promotes leaf germination and growth, increasing the leaf area index (Zhang et al. 2018). This intensifies transpiration and worsens leaf water loss under drought (Qin et al. 2019), which limits phloem carbon transport in the petioles and results in photosynthetic accumulation in leaves (Epron et al. 2019). In contrast, P enrichment (alone or with N) promotes narrower xylem conduits (Faustino et al. 2013, Costa et al. 2021), enhancing embolism resistance (Suissa and Friedman 2021). This hydraulic optimization under P enrichment may reduce reliance on NSC for osmotic adjustment (Jones et al. 2005), inducing less carbon allocation to roots (Li et al. 2016, Jia et al. 2023). In addition, P addition upregulates root aquaporin expression, enhancing root water uptake under drought and maintaining leaf hydration (L.Li et al. 2020, Zeng et al. 2025). This reduces leaf abscisic acid levels and delays drought-induced leaf senescence (Zeng et al. 2025), thereby sustaining photosynthetic activity, phloem loading and promoting assimilate transport from leaves to stems. Such divergent N vs P responses underscore the need to disentangle their individual and interactive impacts on water–carbon relations.

Conventional studies on water–carbon interdependencies have predominantly focused on observable traits such as RWC and NSC concentrations (Rodríguez-Calcerrada et al. 2017, Gomez-Gallego et al. 2022, Duan et al. 2023), while assessments of inter-organ carbon concentration gradient have typically remained simplistic, based mainly on comparing SS (or ST) concentrations across organs (He et al. 2020, L.Lu et al. 2024). However, Yue et al. (2025) recently demonstrated that derived resource allocation attributes—quantifying intra- and inter-organ allocation patterns—provide deeper physiological insights. Specifically, they proposed: (i) intra-organ water–carbon trade-offs: the normalized RWC:SS ratio during drought period reflects tissue-specific reliance on solutes over water for cell turgor maintenance, with a higher normalized ratio indicating greater dependence of the tissue on solutes to sustain cell turgor, while normalized RWC:ST ratio captures trade-offs in cellular volume partitioning between water and ST granules, with a higher ratio indicating a greater cell volume fraction allocated to ST storage relative to water and (ii) inter-organ carbon concentration gradient: differences in SS or ST concentrations exist between organs. During drought, much higher SS or ST concentrations in leaves than twigs may suggest that the transport of newly assimilated carbon is impaired. Notably, Yue et al. (2025) showed that these derived attributes in leaves and twigs outperform absolute concentrations in predicting intraspecific variation in tree growth performance under ambient conditions. However, their utility in belowground modules [e.g. absorptive roots (ARs) vs transport roots (TRs)] remains unexplored.

*Cunninghamia lanceolata* (Lamb.) Hook., a keystone timber species accounting for 36% of global subtropical plantations (Payn et al. 2015, Qu et al. 2022), provides an ideal model system. This study leverages a long-term N and P addition experiment in *C. lanceolata* to: (i) quantify RWC, SS concentration and ST concentration in leaves, twigs, TRs and ARs across control and nutrient addition (N and/or P) treatments during the dry season; (ii) assess treatment effects on derived resource allocation attributes; and (iii) evaluate the relative contributions of these derived attributes to nutrient-induced growth rate variation. We hypothesized that nutrient effects would exert stronger influence on inter-organ carbon concentration gradient than intra-organ water–carbon trade-offs, given N-induced shifts in leaf osmotic potential (increased solute dependence for cell turgor maintenance) (Bucci et al. 2006, Fang et al. 2018) versus branch saturated water content (reduced solute reliance) (Scholz et al. 2007, Yue et al. 2025). We further hypothesized that inter-organ carbon concentration gradient would dominate growth variation, reflecting the established role of longitudinal cellular osmotic gradients (from roots to shoot apex) in predawn meristem rehydration (Zimmermann et al. 2004)—a critical process for subsequent daytime growth.

## Materials and methods

### Site description

The research was conducted at the National Critical Zone Observatory of Qianyanzhou Red Soil Hilly Region (26°44′N, 115°03′E, 102 m a.s.l.) in Jiangxi Province, southern China. This region experiences a typical subtropical East Asian monsoon climate. A distinct seasonal drought period usually occurs from July to October, driven by concurrent high temperatures and reduced precipitation (Song et al. 2020). The study area receives substantial atmospheric N and P deposition, with mean annual inputs of 29 kg N ha^−1^ and 0.56 kg P ha^−1^ (Zhu et al. 2016).

### Experimental design

In November 2011, we established a long-term N and P addition experiment in a 12-year-old *C. lanceolata* plantation, employing a completely randomized block design (Chen et al. 2015). The present study comprised four treatments: control (no nutrient addition), N addition (100 kg N ha^−1^ year^−1^ as NH_4_NO_3_), P addition (50 kg P ha^−1^ year^−1^ as NaH_2_PO_4_) and combined N+P addition (100 kg N + 50 kg P ha^−1^ year^−1^). Each treatment was replicated in three 20 × 20 m plots (*n* = 12 plots total). Pretreatment analysis of diameter at breast height (DBH) confirmed no significant differences among treatments (see Figure S1a available as Supplementary Data at *Tree Physiology* Online). Nutrient additions followed a quarterly schedule: March (30% of annual dose), June (30%), September (20%) and December (20%). For each N and/or P addition treatment, we thoroughly mixed the weighed fertilizers with 8 kg of clean, dry fine sand before manual broadcasting. To ensure uniform distribution, we subdivided each plot into sixteen 5 × 5 m quadrats for precision application. Control plots received sand only following the same protocol.

### Sample collection

In June 2023, we selected 4 healthy *C. lanceolata* trees per plot (12 trees per treatment; 48 trees total) based on plot-mean DBH. Sampling was conducted during mid-summer to mid-autumn (July–October 2023), when seasonal drought constrains tree growth (Zhang et al. 2023), particularly for drought-sensitive *C. lanceolata* (Zang et al. 2021, Wang et al. 2024). Therefore, dry-season sampling can provide mechanistic insight into how internal resource allocation strategies shape tree growth under natural environmental stress. All sampling was initiated at least 7 days after rainfall events. Between 08:00 and 11:00 h, we collected three shallow root samples with intact root systems (first–fifth order) per tree in mid-summer (July), and five sun-exposed lower-mid crown main twigs per tree in early and mid-autumn (September–October). All samples were immediately sealed in plastic bags and stored at 4 °C. Upon return to the laboratory, roots were quickly washed in clean water to remove adhering soil and surface moisture was then immediately and carefully removed using paper towels. This handling step has been previously demonstrated to have negligible effects on root fresh weight (*W_f_*) (Duan et al. 2023). Roots were categorized into ARs (first–second order) and TRs (third–fifth order) (Yin et al. 2020). Twigs were processed by removing bark and phloem tissues. The sampling design yielded 480 leaf/twig samples (4 treatments × 3 plots × 4 trees × 2 organs × 5 replicates) and 288 root samples (4 treatments × 3 plots × 4 trees × 2 modules × 3 replicates). For further analysis, samples were split. For RWC assessment, a minimum of five 2-cm-long segments each of ARs and TRs, three leaves and three 2-cm-long twigs wood segments were selected per sample unit, amounting to no fewer than 15 leaves, twigs, TRs and ARs per tree. Relative water content was determined by recording *W_f_* immediately upon preparation, followed by vacuum-assisted water saturation overnight (Jupa et al. 2016) and measurement of saturated weight (*W_s_*). Dry weight (*W_d_*) was obtained after drying at 65 °C for 72 h. The remaining material was reserved for NSC analysis: samples were enzyme-deactivated at 105 °C for 30 min, dried at 65 °C for 72 h and ground to fine powder using a ball mill (WS-MM301, Germany).

### Resource measurements

Relative water content is a key metric for evaluating drought-induced cellular stress and xylem hydraulic dysfunction (Martinez-Vilalta et al. 2019, Mantova et al. 2023). In this study, we used RWC to characterize organ-level hydration status and water resource pool. Relative water content was calculated as: 


(1) \begin{equation*} \mathrm{RWC}=\frac{W_f-{W}_d}{W_s-{W}_d}\times 100, \end{equation*}

where *W_f_*, *W_s_* and *W_d_* represent fresh, saturated and dry weights, respectively.

Soluble sugar and starch concentrations were determined via the sulfuric acid-anthrone method (Hanson and Møller 1975, Li et al. 2008). For SS extraction, 0.1 g powdered sample was placed in a centrifuge tube and combined with 7 mL deionized water in a water bath at 100 °C for 30 min. The supernatant was collected and the pellet underwent two additional extractions. The remaining residue was dried at 40 °C for 6 h for subsequent ST analysis. Starch hydrolysis involved incubating the dried residue with 3 mL deionized water at 100 °C for 15 min, followed by addition of 2 mL 9.2 mM HClO_4_ for 15 min at room temperature. The supernatant was collected and the pellet was re-extracted with 2 mL 4.6 mM HClO_4_. The concentrations of SS and ST were measured by spectrophotometer (Lambda 25, PerkinElmer, USA) at 620 nm. Starch concentrations were derived by multiplying glucose equivalent concentrations by 0.9. Soluble sugar (or ST) concentrations were calculated on a dry mass basis (%).

### Growth rate

In April 2024, we collected one tree-ring core per tree at breast height using a 5.15 mm diameter increment borer, sampling along the east–west axis. The cores were then air-dried, mounted and carefully polished to expose a clear transverse surface for ring-width analysis. High-resolution digital images were obtained using an Epson Expression 10000 XL flatbed scanner, and tree-ring widths (TRWs) were measured using AutoCAD 2025 software (Autodesk, San Rafael, CA, USA). We verified cross-dating quality through analysis with COFECHA software (Holmes 1983). After 12 years of continuous nutrient additions (2012—23), both P and combined N+P treatments resulted in significantly larger mean DBH compared with control (see Figure S1b available as Supplementary Data at *Tree Physiology* Online). To reflect the long-term nutrient effects on tree growth, we derived annual growth rates from TRW measurements for the period 2019 to 2023. The growth rate (in mm per year) was calculated as:


(2) \begin{equation*} \mathrm{Growth}\ \mathrm{rate}=\frac{\sum{\mathrm{TRW}}_i}{n}, \end{equation*}

where TRW_*i*_ is the tree-ring width from 2019 to 2023, *n* is 5 years.

### Calculation of derived resource allocation attributes

Intra-organ water–carbon trade-offs were quantified following Yue et al. (2025). First, we ranked the values of RWC, SS concentration and ST concentration within each organ type across all samples (48 individuals × 5 replicates for leaves and twigs; 48 individuals × 3 replicates for TRs and ARs), with smaller ranks indicating higher original values. The degree of the RWC and SS trade-off was quantified as *ln*(*RWC*_rank_/*SS*_rank_), where *RWC*_rank_ and *SS*_rank_ are the ranks of RWC and SS, respectively. *ln*(*RWC*_rank_/*SS*_rank_) > 0 suggests higher dominance of SS over RWC, *ln*(*RWC*_rank_/*SS*_rank_) < 0 suggests higher dominance on RWC over SS and *ln*(*RWC*_rank_/*SS*_rank_) = 0 suggests RWC and SS match each other. The same approach was applied for RWC and ST trade-off using *ln*(*RWC*_rank_/*ST*_rank_). We used rank-based attributes because raw SS and ST show substantial heterogeneity across individuals. Starch, in particular, displays typical zero inflation and spans multiple orders of magnitude. Rank transformation provides a simple, non-parametric way to handle these distributional issues without the need to assume normality or to arbitrarily treat zeros.

To evaluate inter-organ carbon concentration gradient, we calculated SS (or ST) concentration difference between leaves and twigs [*SS_leaf-twig_* (or *ST_leaf-twig_*)] by subtracting the respective values in leaves from twigs. Direct subtraction is straightforward and easier to interpret because the units and meanings of the SS (or ST) are the same. *SS_leaf-twig_* > 0 means higher SS concentrations in leaves than in twigs; *SS_leaf-twig_* < 0 means lower concentrations in leaves than in twigs and *SS_leaf-twig_* = 0 means equal concentrations in leaves and twigs. The same approach was applied between ARs and TRs [*SS_AR-TR_* (or *ST_AR-TR_*)]. These attributes capture both the magnitude and direction of inter-organ carbohydrate concentration gradient. Table 1 summarizes all resource attributes, including traditional resource attributes and derived resource allocation attributes.

**Table 1:** Abbreviations, descriptions and units of attributes studied.

Attribute	Abbreviation	Description	Unit
Organ hydration status	RWC	Relative water content	%
Observable resource concentrations	SS concentration	Soluble sugar concentration, serving as an accessible metabolic currency and providing osmoprotection	%
	ST concentration	Starch concentration, a long-term carbon reserve	%
Intra-organ water–carbon trade-offs	*ln*(*RWC*_rank_/*SS*_rank_)	Water–SS trade-offs within organ. Higher values during drought indicate higher dominance of SS over RWC and likely greater dependence of cell turgor maintenance on solutes	—
	*ln*(*RWC*_rank_*/ST*_rank_)	Water–ST trade-offs within organ. Higher values indicate higher dominance of ST over RWC and a greater cell volume fraction for starch storage	—
Inter-organ carbon concentration gradient	*SS_leaf-twig_*	SS concentration gradient between leaves and twigs. Positive values indicate higher SS concentration in leaves than in twigs	%
	*SS_AR-TR_*	SS concentration gradient between ARs and TRs. Positive values indicate higher SS concentration in ARs than in TRs	%
	*ST_leaf-twig_*	ST concentration gradient between leaves and twigs. Positive values indicate higher ST concentration in leaves than in twigs	%
	*ST_AR-TR_*	ST concentration gradient between ARs and TRs. Positive values indicate higher ST concentration in ARs than in TRs	%

### Data analysis

Prior to statistical analysis, extreme outliers were excluded. Outliers were defined as values deviating more than two standard deviations from the sample mean. Normality and homogeneity of variance were examined across all data using the Kolmogorov–Smirnov test and Levene’s test, respectively. For data adhering to normal distribution and homogeneous variance, treatment effects were assessed using one-way analysis of variance (ANOVA) followed by Tukey’s HSD. Otherwise, the non-parametric Tamhane’s T2 test was applied.

To analyse the relationships between growth rates and resource attributes, the mean resource attributes for each individual tree were calculated. Linear and quadratic polynomial regressions were applied to screen for resource attributes significantly associated with growth rate. The accuracy and predictive power of the linear and quadratic polynomial fits were evaluated using fivefold cross-validation based on root mean square error (RMSE), mean absolute error (MAE) and adjusted *R*^2^, where lower RMSE and MAE and higher adjusted *R*^2^ indicate better model performance (Champion et al. 2018, Fan et al. 2018, Liao et al. 2025).

We note that the absolute values of rank-based attributes for intra-organ water–carbon trade-offs are study-specific. However, the primary value of our index is not to provide a universal numerical score, but to reveal stable biological relationships. To demonstrate this, we performed 500 bootstrap iterations with replacement on 48 trees (each time recalculating all ranks and the index) and examined the robustness of the relationships between intra-organ water–carbon trade-offs and growth rate using 95% confidence intervals.

Significant attributes were then used to calculate the contribution to the growth rate. When analyzing direct observable resource concentrations alone, hierarchical partitioning was employed to quantify their relative contributions to growth rates (Yue et al. 2025). For analyses incorporating derived resource allocation attributes, a generalized additive model (GAM) was used to evaluate the contribution of each attribute to growth rate, accommodating nonlinear responses (Lai et al. 2024, Kindermann et al. 2025, Song et al. 2025). The GAM used a Gaussian family with an identity link function. The smooth term was specified using thin-plate regression splines as the basis function, with the basis dimension *k* = 9, and smoothing parameters were estimated using generalized cross-validation. In addition, we conducted basic diagnostics for the GAM, covering residual analysis, concurvity assessment and overfitting checks. Normality, variance homogeneity test and one-way ANOVA were conducted in SPSS version 26.0 (IBM Corp, Armonk, NY, USA). Other statistical analyses, including hierarchical partitioning, GAM analyses, fivefold cross-validation and bootstrap resampling, were conducted in R v.4.4.1.

## Results

### Effects of nutrient addition on traditional resource attributes

**Figure 1:**
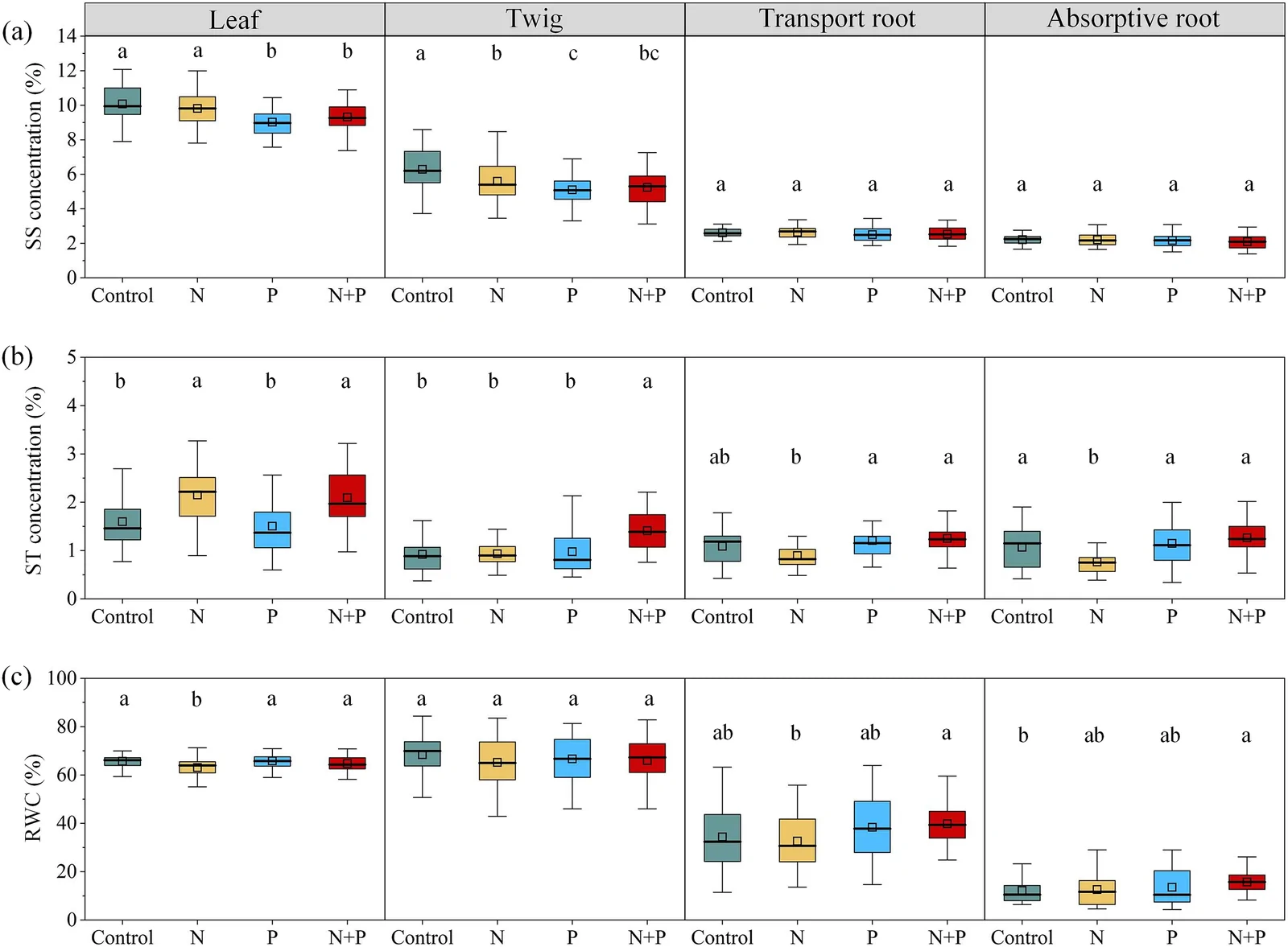
Nutrient effects on (a) SS concentration, (b) ST concentration and (c) RWC of leaves, twigs, TRs and ARs. Normality and homogeneity of variance are shown in Tables S1 and S2 available as Supplementary Data at *Tree Physiology* Online, respectively. Box plots show mean values (black squares) and median values (black solid lines). N, N addition; P, P addition; N+P, combined N+P addition. Different letters indicate significant differences between treatments (*P* < 0.05).

Nutrient additions exerted stronger influences on carbohydrate concentrations (SS and ST concentrations) than on RWC across all plant organs (Figure 1). Nitrogen addition significantly elevated leaf ST concentrations by 34.6%, while reducing twig SS, and absorptive root ST concentrations by 11.0 and 28.4%, relative to the control (all *P* < 0.05) (Figure 1a and b). Phosphorus addition decreased SS concentrations in both leaves (10.5%) and twigs (18.9%) (both *P* < 0.05) (Figure 1a). The combined N+P addition increased ST concentrations in leaves (31.1%) and twigs (53.4%), while decreasing SS concentrations in leaves (7.5%) and twigs (16.7%) (all *P* < 0.05) (Figure 1a and b). Nitrogen addition reduced leaf RWC by 3.9%, while combined N+P addition increased RWC in ARs by 29.1% compared with control (both *P* < 0.05) (Figure 1c).

### Effects of nutrient addition on intra-organ water–carbon trade-offs

**Figure 2:**
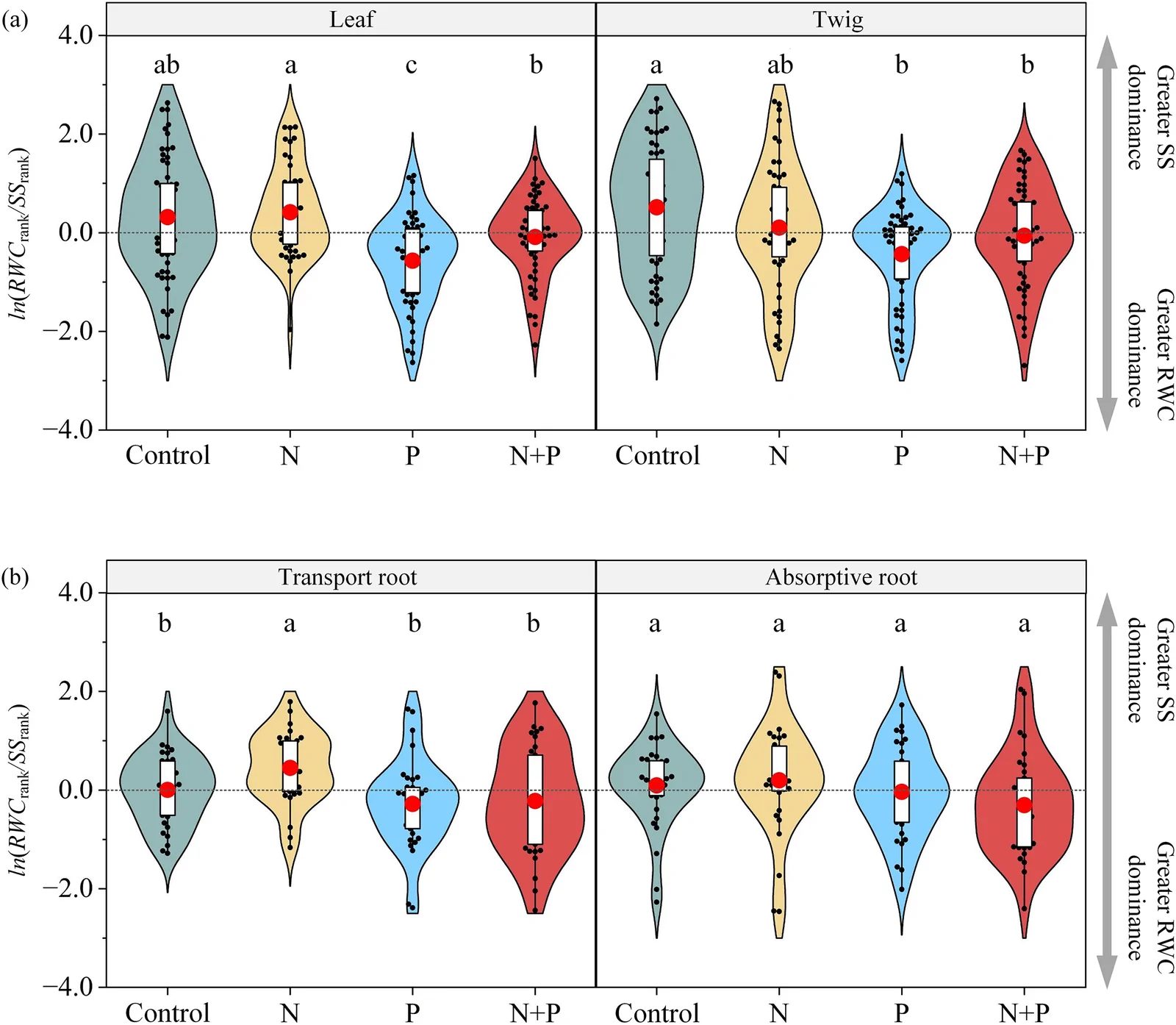
Nutrient effects on *ln*(*RWC*_rank_/*SS*_rank_) within organs. (a) Leaves and twigs and (b) TRs and ARs. *ln*(*RWC*_rank_/*SS*_rank_) > 0 suggests greater SS dominance over RWC, *ln*(*RWC*_rank_/*SS*_rank_) < 0 suggests greater RWC dominance over SS and *ln*(*RWC*_rank_/*SS*_rank_) = 0 suggests the RWC and SS match each other (dashed line). Normality and homogeneity of variance are shown in Tables S1 and S2 available as Supplementary Data at *Tree Physiology* Online, respectively. Box plots show mean values (red circles). Black dots represent samples of each organ. N, Nitrogen addition; P, P addition; N+P, combined N+P addition. Different letters indicate significant differences between treatments (*P* < 0.05).

Nutrient treatments significantly altered the trade-offs between RWC and SS (Figure 2). Relative to the control, P addition decreased *ln*(*RWC*_rank_/*SS*_rank_) values in both leaves and twigs (both *P* < 0.05), and combined N+P addition decreased this parameter only in twigs (*P* < 0.05) (Figure 2a), demonstrating enhanced RWC dominance. Specifically, P addition reversed the control’s SS dominance to RWC dominance, while combined N+P addition balanced this relationship, with mean values approaching zero (indicating a relative equilibrium). Root systems exhibited distinct responses. Nitrogen addition elevated *ln*(*RWC*_rank_/*SS*_rank_) in TRs relative to the control (*P* < 0.05) (Figure 2b), establishing SS dominance. In contrast, P and combined N+P additions promoted RWC dominance in these roots compared with N addition. Absorptive roots showed no significant *ln*(*RWC*_rank_/*SS*_rank_) changes versus control (Figure 2b).

**Figure 3:**
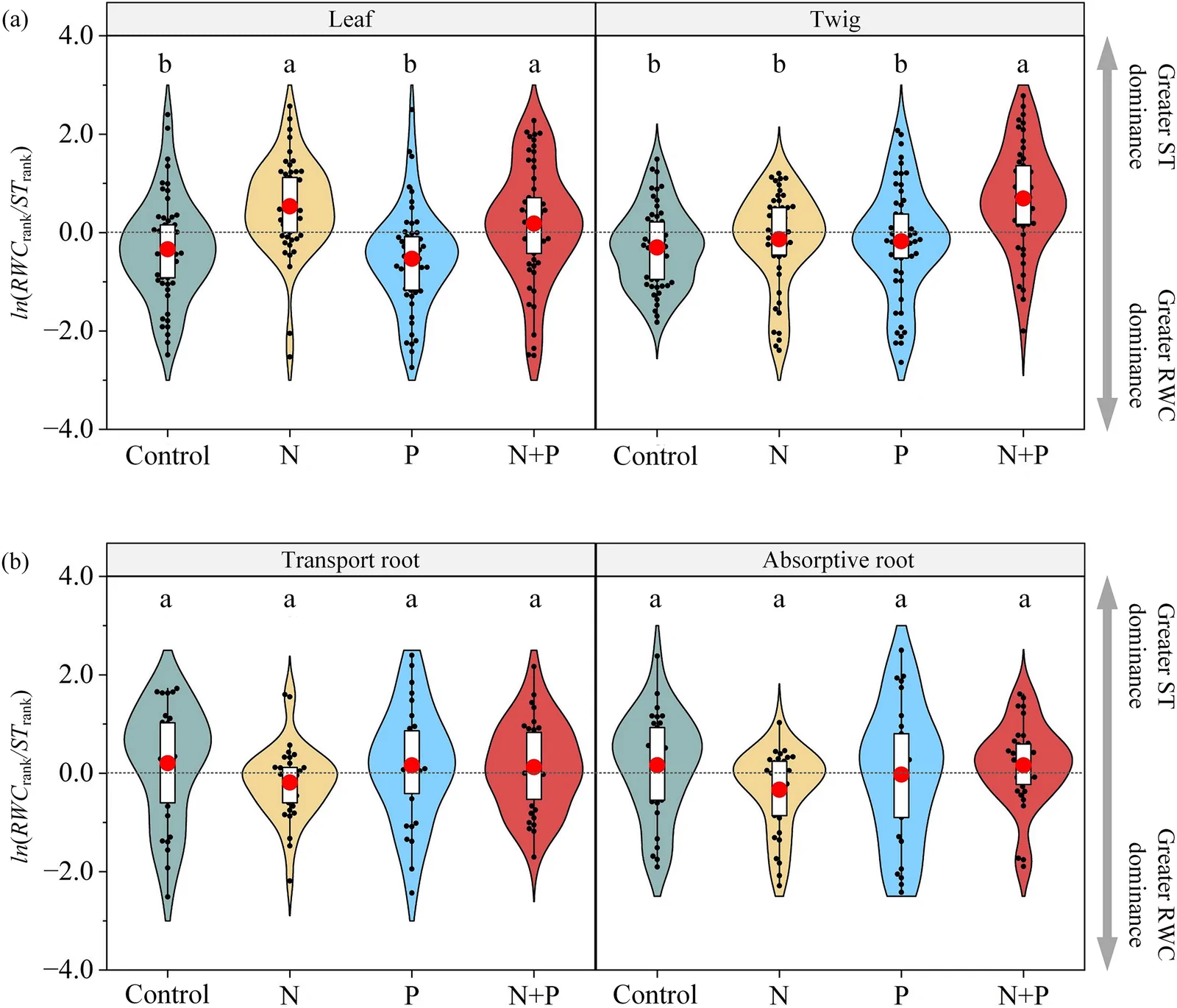
Nutrient effects on *ln*(*RWC*_rank_/*ST*_rank_) within organs. (a) Leaves and twigs and (b) TRs and ARs. *ln*(*RWC*_rank_/*ST*_rank_) > 0 suggests greater ST dominance over RWC, *ln*(*RWC*_rank_/*ST*_rank_) < 0 suggests greater RWC dominance over ST and *ln*(*RWC*_rank_/*ST*_rank_) = 0 suggests the RWC and ST match each other (dashed line). Normality and homogeneity of variance are shown in Tables S1 and S2 available as Supplementary Data at *Tree Physiology* Online, respectively. Box plots show mean values (red circles). Black dots represent samples of each organ. N, N addition; P, P addition; N+P, combined N+P addition. Different letters indicate significant differences between treatments (*P* < 0.05).

Nutrient addition effects on the trade-offs between RWC and ST were organ-specific, with significant impacts only in leaves and twigs (Figure 3). Nitrogen addition increased *ln*(*RWC*_rank_/*ST*_rank_) in leaves relative to the control (*P* < 0.05) (Figure 3a), shifting from RWC dominance to ST dominance. Notably, twigs exhibited positive *ln*(*RWC*_rank_/*ST*_rank_) values under combined N+P addition (*P* < 0.05) (Figure 3a), representing the sole treatment with clear ST predominance over RWC. While P addition showed no significant effects relative to control for any organ (Figure 3), it significantly reduced leaf *ln*(*RWC*_rank_/*ST*_rank_) values compared with N addition (*P* < 0.05) (Figure 3a), counteracting N-induced ST dominance and restoring water resource prevalence.

### Effects of nutrient addition on inter-organ carbon concentration gradient

**Figure 4:**
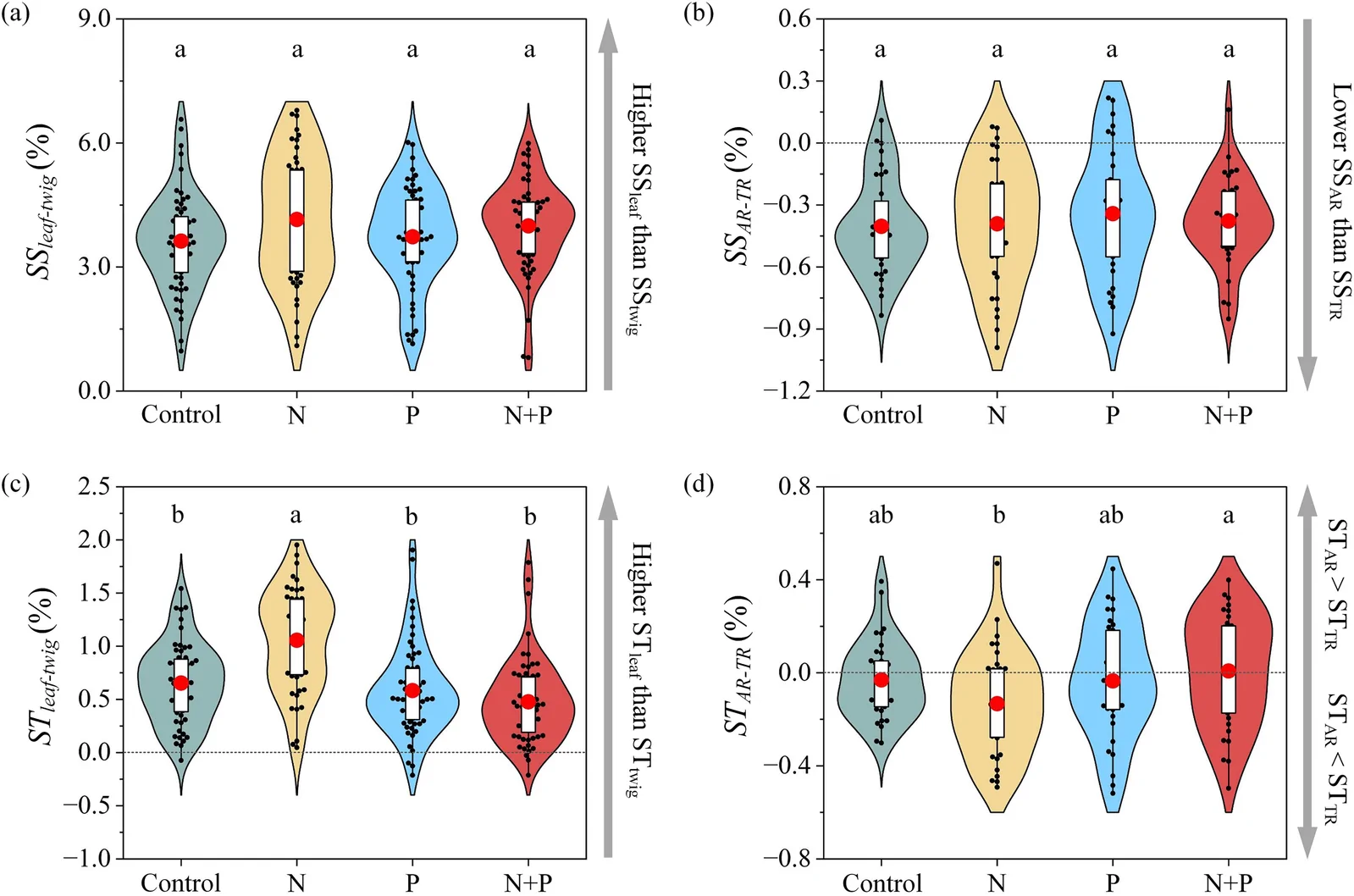
Nutrient effects on inter-organ carbon concentration gradient. (a) Soluble sugar concentration gradient between leaves and twigs (*SS_leaf-twig_*); (b) SS concentration gradient between ARs and TRs (*SS_AR-TR_*); (c) ST concentration gradient between leaves and twigs (*ST_leaf-twig_*); and (d) ST concentration gradient between ARs and TRs (*ST_AR-TR_*). *SS_leaf-twig_* > 0 suggests that SS concentrations in leaves are higher than in twigs, *SS_leaf-twig_* < 0 suggests that SS concentrations in leaves are lower than in twigs and *SS_leaf-twig_* = 0 suggests equal SS concentrations between leaves and twigs (dashed line). The meaning of *SS_AR-TR_*, *ST_leaf-twig_* and *ST_AR-TR_* are similar to that of *SS_leaf-twig_*. Normality and homogeneity of variance are shown in Tables S1 and S2 available as Supplementary Data at *Tree Physiology* Online, respectively. Box plots show mean values (red circles). Black dots represent samples of each organ. N, N addition; P, P addition; N+P, combined N+P addition. Different letters indicate significant differences between treatments (*P* < 0.05).

Nitrogen addition uniquely influenced the concentration gradient of ST between leaves and twigs compared with P and combined N+P treatments (Figure 4). Although ST concentrations remained higher in leaves than twigs, N addition significantly amplified the magnitude of concentration gradient between these organs. Specifically, N addition elevated *ST_leaf-twig_* values compared with the control (*P* < 0.05) (Figure 4c), indicating an enhanced accumulation of these concentrations in leaves relative to twigs. In contrast, nutrient addition did not significantly alter the SS (or ST) concentration gradient between ARs and TRs compared with the control (Figure 4b and d).

### Relationship between resource attributes and growth rates

**Figure 5:**
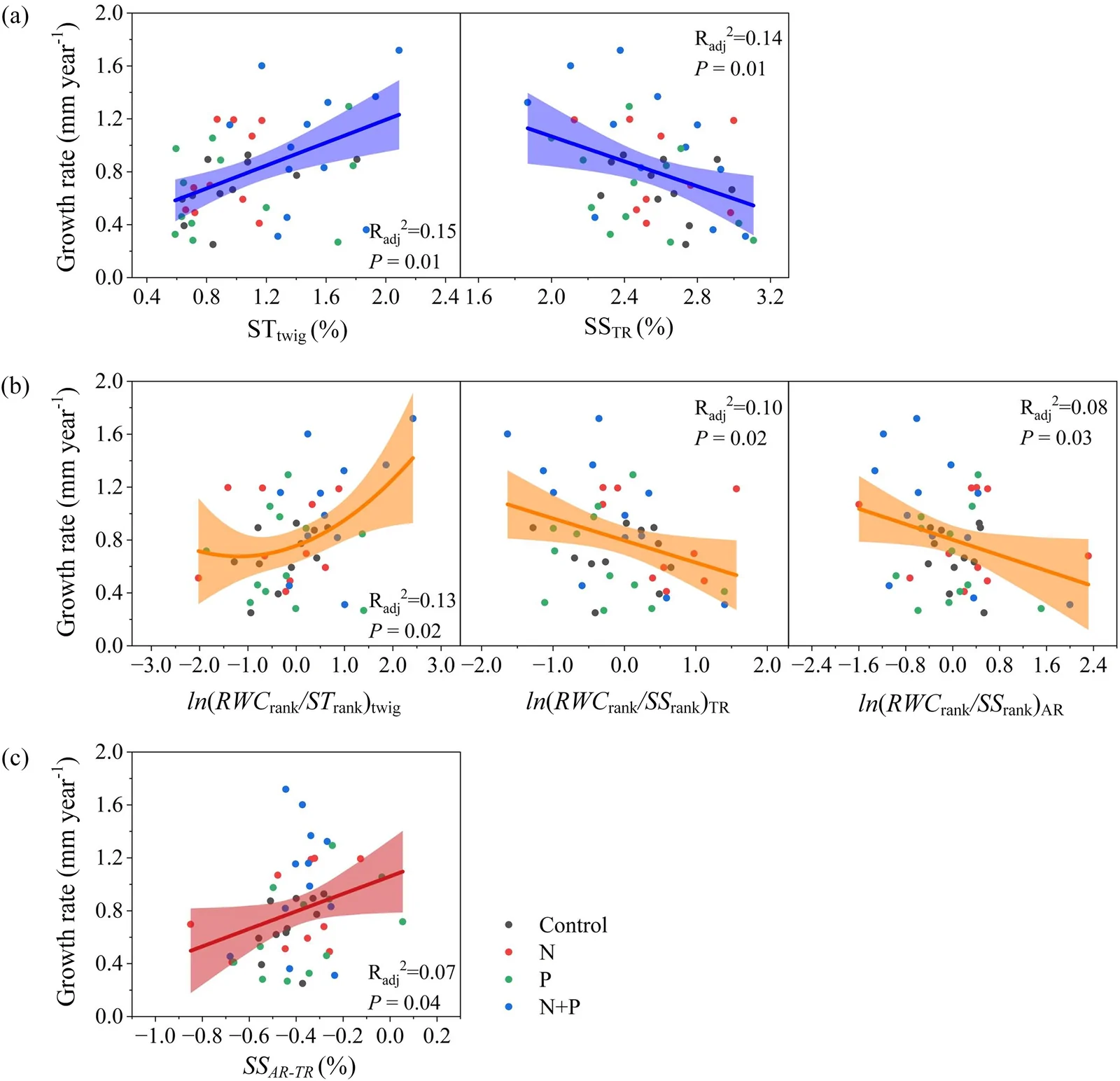
Relationships between tree growth rate and (a) observable resource concentrations, (b) intra-organ water–carbon trade-offs and (c) inter-organ carbon concentration gradient. The optimal fitting model for each relationship is provided in Table S3 available as Supplementary Data at *Tree Physiology* Online. Non-significant relationships are presented in Table S4 available as Supplementary Data at *Tree Physiology* Online. Shaded areas represent the 95% confidence interval. *R*_adj_^2^ is the adjusted *R*^2^.

Resource attributes of twigs and roots showed significant correlations with tree growth rates (Figure 5, see Table S4 available as Supplementary Data at *Tree Physiology* Online). Among observable resource concentrations, ST_twig_ was positively correlated with growth rate (*P* = 0.01), while SS_TR_ showed a negative correlation (*P* = 0.01) (Figure 5a), indicating faster-growing individuals maintained higher ST concentrations in twigs and lower SS concentrations in TRs.

Intra-organ water–carbon trade-offs revealed a positive relationship between twig *ln*(*RWC*_rank_/*ST*_rank_) and growth rate (*P* = 0.02) (Figure 5b), indicating that ST dominance over RWC in twigs promoted growth. Conversely, negative correlations emerged for both TRs (*P* = 0.02) and ARs (*P* = 0.03) (Figure 5b), with belowground RWC dominance over SS favoring faster growth. To assess the stability of the rank-based approach, we performed bootstrap resampling (500 iterations) (see Figure S2 available as Supplementary Data at *Tree Physiology* Online). Despite the 95% confidence interval for twig *ln*(*RWC*_rank_/*ST*_rank_) marginally including zero [(−0.003, 0.22)], the coefficient estimates were consistently positive across replicates (concentrated between 0.05 and 0.15), supporting the robustness of the directionality. The other two relationships showed stable negative trends, with confidence intervals entirely below zero [(−0.31, −0.08) and (−0.32, −0.05)]. These results indicate that the rank-based method yields consistent directional trends, even when some point estimates nearing zero.

At the inter-organ level, reduced SS concentration gradient between ARs and TRs (*SS_AR-TR_* value closer to zero within negative ranges) were linked to faster growth rates (*P* = 0.04) (Figure 5c).

**Figure 6:**
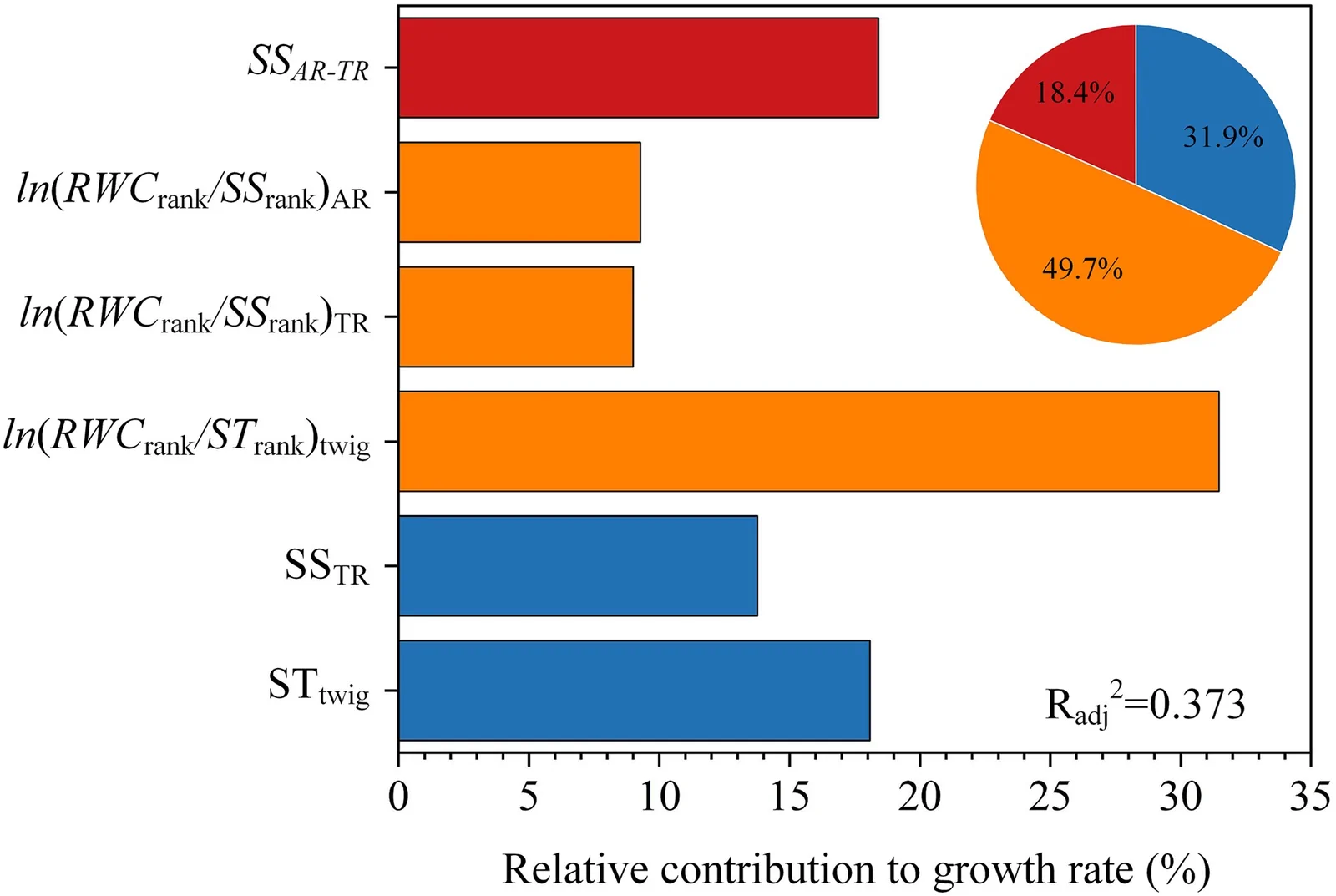
Generalized additive model quantifying the relative contributions of key observable resource concentrations, intra-organ water–carbon trade-offs and inter-organ carbon concentration gradient to growth rate variation. The pie chart illustrates the cumulative contribution of each type of resource attribute. Blue represents observable resource concentrations, yellow represents intra-organ water–carbon trade-offs and red represents inter-organ carbon concentration gradient. Residual plots showed a good model fit (see Figure S4 available as Supplementary Data at *Tree Physiology* Online). Concurvity (‘worst-case’ = 0.72) was below the 0.8 threshold (Soto et al. 2025). No overfitting (edf = 4.87 < k = 9.00) or under-smoothing (k-index = 1.07 > 1, *P* = 0.66) was detected. *R*_adj_^2^ is the adjusted *R*^2^.

Although individual resource attribute individually explained only a small fraction of growth variation (low adjusted *R*^2^), their combined influence was substantial (Figures 5 and 6, see Figure S3 available as Supplementary Data at *Tree Physiology* Online). When only observable resource concentrations were considered, hierarchical partitioning revealed that these attributes contributed 26.0% to the growth rate (See Figure S3 available as Supplementary Data at *Tree Physiology* Online). After adding the derived resource allocation attributes, GAM analysis indicated that the overall contribution of resource attributes to growth rate increased to 37.3% (a 43.5% improvement) (Figure 6). The GAM revealed that derived resource allocation attributes exhibited 2.1-fold greater explanatory power for growth variation compared with traditional observable concentrations (68.1 vs 31.9%). Notably, intra-organ water–carbon trade-offs collectively explained 49.7% of the total variance in growth performance, demonstrating their dominant predictive role. Among these, twig *ln*(*RWC*_rank_/*ST*_rank_) emerged as the single strongest predictor, accounting for 31.5% of the explanatory power for growth rate variation.

## Discussion

**Figure 7:**
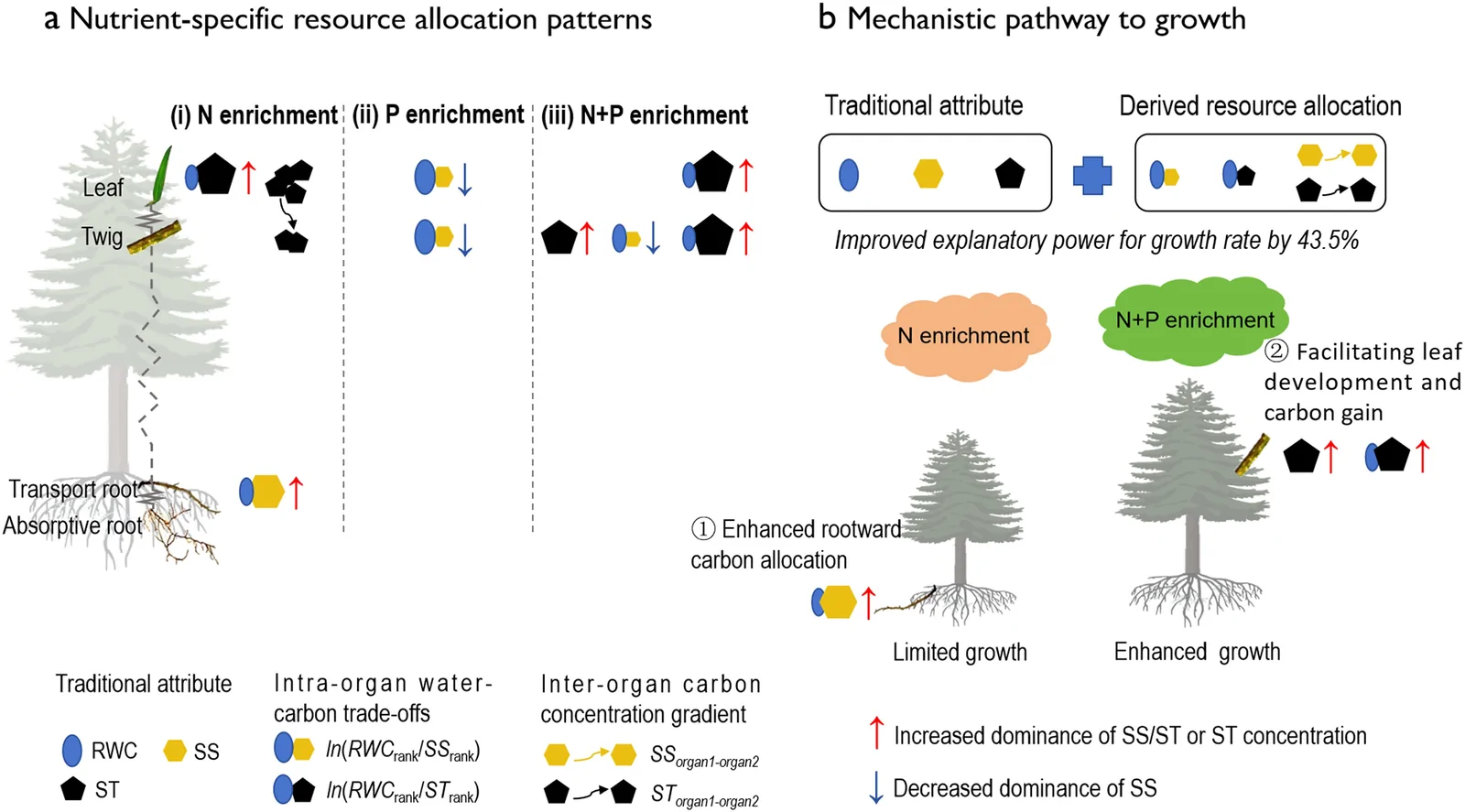
Nutrient-driven reorganization of water–carbon resource allocation in *C. lanceolata* and its implications for growth rate. (a) Nutrient-specific shifts in resource allocation: (i) N addition enhanced SS dominance in TRs, increased ST dominance in leaves and strengthened the ST concentration gradient; (ii) P addition reduced SS dominance in leaves and twigs; and (iii) combined N+P addition reduced SS dominance in twigs and facilitated ST-dominated storage in both leaves and twigs. (b) Mechanistic pathways linking resource allocation to growth: incorporating derived attributes into traditional resource attributes increased explanatory power for growth rate by 43.5%. Nutrient enrichment modulates growth through resource allocation, with two distinct pathways: ① N-induced sink strength dominance in TRs [higher *ln*(*RWC*_rank_/*SS*_rank_)], reflecting shifted carbon allocation to roots (Huang et al. 2021b) at the potential expense of stem growth; ② N+P-induced ST accumulation in twigs [higher *ln*(*RWC*_rank_/*ST*_rank_)], facilitating new leaf development and establishing a positive feedback loop via enhanced photosynthesis and whole-plant carbon assimilation (Meng et al. 2015).

While previous studies have primarily examined nutrient-mediated changes in observable resource concentrations (e.g. SS and ST concentrations across tree organs) (Li et al. 2016, W.Li et al. 2020, Ye et al. 2023), our study advances this paradigm by demonstrating that nutrient enrichment fundamentally alters derived resource allocation attributes (Figure 7a). Specifically, we showed that nutrient enrichment modified both intra-organ water–carbon trade-offs and inter-organ carbon concentration gradient. The intra-organ trade-offs were quantified by two normalized ratios—*ln*(*RWC*_rank_/*SS*_rank_) and *ln*(*RWC*_rank_/*ST*_rank_)—which reflect the relative dominance of water versus carbohydrate resources within tissue. Crucially, the twig *ln*(*RWC*_rank_/*ST*_rank_) emerged as the strongest growth predictor among those examined (Figure 6). These findings highlight the potential of derived resource allocation attributes as novel diagnostic tools for predicting adaptive responses under global change scenarios.

### Nutrient-specific modulation of resource allocation: contrasting N, P and combined N+P additions effects

Contrary to our initial hypothesis, nutrient addition exerted stronger effects on intra-organ water–carbon trade-offs rather than inter-organ carbon concentration gradient (Figures 2–4 and 7a). Nitrogen addition enhanced the SS resource dominance over RWC in TRs (Figure 2b), a response likely mediated by multiple interacting physiological mechanisms. First, N addition could elevate root N content (Ma et al. 2021), particularly under concurrent drought conditions (Zhang et al. 2024), which requires energy-intensive conversion of inorganic N to organic compounds (Santiago et al. 2012, Krapp 2015). Therefore, in trees from the N addition treatment increased SS dominance in TRs was required to support N assimilation. Second, *C. lanceolata* is generally colonized by arbuscular mycorrhizal (AM) fungi (Cao et al. 2020). Nitrogen-induced P demand enhances carbon allocation to AM fungi (Wu et al. 2024), increasing fungal colonization and root carbon sink strength—a process that would further elevate SS demand in ARs. Third, N addition could increase transport root tracheid diameter (Wang et al. 2017), potentially elevating the risk of xylem embolism during drought. Therefore, enhancement of SS in TRs may contribute to maintenance of cell turgor and hydraulic function under water stress. Intriguingly, N addition reversed the dominant resource in leaves, shifting from RWC dominance to ST dominance (Figure 3a). This shift may represent an induced defense strategy, as N enrichment improves leaf palatability to herbivores particularly under drought conditions (Taboada et al. 2016, Zhou et al. 2020, Ma et al. 2025), whereas increased ST dominance could support sustained production of defensive volatile organic compounds (Hao et al. 2024). At the inter-organ level, N addition amplified the concentration gradient of ST from leaves to twigs (Figure 4c). This pattern likely reflects both impaired carbohydrate export (due to N-induced increases in leaf xylem embolism risk and petiole phloem sap viscosity, Epron et al. 2019, Jin et al. 2020) and enhanced transpiration-driven solute flux toward leaves (Lu et al. 2018, Tixier et al. 2018). The accumulation of carbohydrates in leaves may in turn drive the conversion of SS to ST for storage, potentially contributing to the observed ST concentration gradient.

Phosphorus and combined N+P additions selectively modified intra-organ water–carbon trade-offs in aboveground organs. Phosphorus addition reduced SS dominance over RWC in leaves and twigs, and combined N+P addition only decreased SS dominance in twigs (Figure 2a), reflecting decreased reliance on osmotic adjustment for turgor maintenance during drought. This response appears to be mediated by two complementary mechanisms. First, P and combined N+P additions significantly increased stem diameter (see Figure S1b available as Supplementary Data at *Tree Physiology* Online), enhancing stem water storage capacity (Yan et al. 2023), thereby buffering transpirational water losses. Second, P accumulation may improve cellular dehydration resistance through physicochemical modifications of the protoplast, including increased hydration of protoplast colloids and reduced cell wall thickness with a consequent increase in flexibility, which collectively increase protoplast viscosity and elasticity (Khan et al. 2024, Gan et al. 2025). These modifications would reduce the reliance on solute accumulation for turgor maintenance during water stress.

Combined N+P addition uniquely shifted resource allocation priorities from RWC to ST dominance in twigs (Figure 3a), a response likely reflecting synergistic nutrient effects on photosynthetic performance. Compared with single-nutrient treatments, combined N+P addition could increase leaf chlorophyll content (Tao et al. 2022), enhancing daytime carbon assimilation. The resulting photosynthate is transiently stored as ST in leaves, followed by nocturnal conversion to SS for phloem transport to twigs (Smith and Zeeman 2020). In twigs, these carbohydrates were subsequently reconverted to ST for stable storage, collectively explaining the observed ST dominance across both leaves and twigs.

### Resource allocation mediates nutrient-induced growth variation in trees

Despite the low individual explanatory power of individual resource attribute (low adjusted *R*^2^; Figure 5), their collective effect was substantial, yielding an overall adjusted *R*^2^ of 0.373 for growth rate (Figure 6, see Figure S3 available as Supplementary Data at *Tree Physiology* Online). This indicates that these resource allocation attributes possess important physiological significance for explaining variations in growth rate. Three derived attributes in belowground organs—*ln*(*RWC*_rank_/*SS*_rank_)_TR_, *ln*(*RWC*_rank_/*SS*_rank_)_AR_ and *SS_AR-TR_*—were significantly correlated with growth rate (Figure 5b and c), extending previous findings from non-fertilized conditions that were restricted to aboveground organs (Yue et al. 2025). Transport roots consistently exhibited higher SS concentrations than ARs (Figure 1a), reflecting their dual role in resource translocation and storage (Sanaei et al. 2025). While negative *SS_AR-TR_* values (SS_AR_ is lower than SS_TR_) theoretically facilitate cell osmotic pressure–coupled longitudinal water flow from ARs to TRs (Zimmermann et al. 2004), our results paradoxically showed that less negative *SS_AR-TR_* (indicating potentially reduced SS-dependent osmotic gradients) correlated with faster growth (Figure 5c). This suggests that increased SS flux from transport to ARs may confer two key advantages: (i) enhanced osmotic adjustment capacity in ARs (Voothuluru et al. 2024), improving nocturnal root-pressure-driven radial water uptake (Zimmermann et al. 2004) and (ii) stimulation of AM fungal hyphal network development, thereby facilitating soil-water delivery to host plants (Kakouridis et al. 2022, Tang et al. 2024). Contrary to our second hypothesis, intra-organ water–carbon trade-offs—particularly twig *ln*(*RWC*_rank_/*ST*_rank_)—emerged as the strongest predictors of growth rate variability (Figure 6). Higher ST dominance in twigs was associated with faster growth (Figure 5b), likely because it provides both energetic and defensive benefits. First, greater ST dominance in twigs facilitates rapid post-drought recovery, supporting regrowth after drought relief (Huang et al. 2021*a*, Silvestro et al. 2024). Second, as twigs in our study were sampled in early and mid-autumn, ST stored primarily during this period is essential for the spring’s bud flush and shoot elongation (Regier et al. 2010). Third, given the frequent occurrence of winter low temperatures in subtropical regions (Yin et al. 2025), stored ST preferentially aids trees cope with such conditions through rapid conversion to SS, which enhances low-temperature tolerance and prevents growth inhibition in the subsequent year (Chen et al. 2025). However, future studies should examine whether considering glucose and fructose gradients (with their stronger osmotic effects) might alter the relative explanatory power of intra- versus inter-organ resource allocation.

Collectively, these resource allocation–growth relationships provide a physiological explanation for the contrasting growth responses to N and N+P enrichment in tropical or subtropical forests, beyond established mechanisms such as soil and plant tissue stoichiometry (Fan et al. 2015, Bu et al. 2021, Liu et al. 2024), morphological traits of plant tissues (Tripathi and Raghubanshi 2014), and herbivore-mediated effects (Andersen et al. 2010). Nitrogen enrichment increases root N metabolic demands (Santiago et al. 2012, Krapp 2015), potentially leading to redirected allocation of SS toward roots (Huang et al. 2021*b*). This shift enhanced SS dominance over RWC in TRs (Figure 2b), a pattern that may occur at the expense of stem growth. Although no significant correlation was detected between *SS_leaf-twig_* and growth rate in this study, this parameter exhibited an increasing trend under N addition (a 14% increase), indicating unusually high accumulation of SS in leaves. Yue et al. (2025) reported that, under non-fertilized conditions, higher *SS_leaf-twig_* limited the growth of *C. lanceolata*. The potential reason is that the phloem carbon transport capacity of petioles declines during the dry season (Epron et al. 2019), blocking the carbon translocation from leaves to twigs. This may lead to insufficient carbon supply for stem and thereby constrain tree growth. Therefore, we recommend that further work with broader geographic coverage and larger sample sizes be conducted to fully unravel the mechanisms driving N-mediated tree growth limitation, particularly in relation to carbon concentration gradient. In contrast, combined N+P addition enhanced ST concentrations in twigs (Figure 1b) and thus ST dominance over RWC (Figure 3a). As twigs (particularly current-year terminal twigs) serve as critical hubs for leaf emergence (Meng et al. 2015), their ST-dominated resource storage likely facilitates new leaf development creating a positive feedback loop through enhanced photosynthetic capacity and whole-plant carbon gain.

### Towards mechanistic prediction of plant responses to global change: the role of resource allocation

Our demonstration that derived attributes effectively predict tree growth responses to N and P deposition (Figures 6 and 7b) highlights their potential for understanding broader global change impacts. For instance, warming reduces leaf SS concentrations while maintaining RWC (Balfagón et al. 2019, Du et al. 2020, Ouyang et al. 2024, Arciszewski et al. 2025), potentially decreasing SS dominance. Conversely, drought decreases leaf RWC while enhancing SS concentrations (Du et al. 2020, Duan et al. 2023), potentially increasing SS dominance. Critically, these stressors differently alter inter-organ concentration gradient patterns. Warming disproportionately reduces leaf versus stem SS concentrations, whereas drought generates opposing allocation patterns (increased leaf but decreased stem SS concentrations) (Du et al. 2020)—suggesting fundamental reorganization of both carbon transport and osmotic gradient-related solute gradients. Future cross-species validation of these response patterns could establish resource allocation as a robust paradigm for predicting tree performance under multifactorial global change scenarios, particularly where traditional resource attributes have proven inadequate (Hartmann et al. 2020, Piper et al. 2022).

Our results revealed homeostatic maintenance of SS and ST concentration gradient between ARs and TRs in *C. lanceolata* under nutrient enrichment (Figure 4b and d), indicating conserved carbohydrate allocation pattern between ARs and TRs in this species. However, emerging comparative evidence highlights substantial interspecific variation in root resource allocation under global change. For instance, drought elicits divergent responses: *Juglans mandshurica* Maxim. exhibited parallel SS decline in both absorptive (first order) and transport (fifth order) roots (Ji et al. 2023), while *Fraxinus mandshurica* Rupr. maintained transport root SS despite reductions in ARs (Ji et al. 2021). These contrasting patterns suggest species-specific divergence in belowground resource gradient plasticity, potentially reflecting different ecological strategies for stress adaptation. Future studies should address two critical knowledge gaps: (i) phylogenetic patterns in root resource allocation plasticity across plant functional types and (ii) how interspecific variation in belowground resource allocation mediates ecological coexistence under changing environments. Resolving these questions could unify our understanding of whether conserved or plastic resource allocation strategies determine species-specific responses to global change.

## Conclusions

Our study demonstrates that nutrient enrichment fundamentally reorganizes internal resource (water and carbon) allocation strategies in *C. lanceolata*, with cascading effects on derived resource allocation attributes. We demonstrate three key nutrient-specific responses: (i) N addition promoted TR *ln*(*RWC*_rank_/*SS*_rank_) while enhancing leaf *ln*(*RWC*_rank_/*ST*_rank_), amplifying *ST_leaf-twig_*; (ii) P addition reduced leaf and twig *ln*(*RWC*_rank_/*SS*_rank_); and (iii) combined N+P addition reduced twig *ln*(*RWC*_rank_/*SS*_rank_), while increasing leaf and twig *ln*(*RWC*_rank_/*ST*_rank_). These reorganization patterns had direct growth consequences. While traditional resource concentrations explained 26.0% of growth variation, incorporating derived attributes increased explanatory power by 43.5%, accounting for 37.3% of the total variance. Notably, N enrichment enhanced SS dominance over RWC in TRs, a pattern that may occur at the expense of stem growth. In contrast, N+P enrichment promoted the ST accumulation in twigs, leading to ST dominance over RWC that was correlated with higher productivity. Collectively, these findings provide resource-allocation insights into why N-only enrichment often fails to stimulate growth in subtropical forests whereas N+P co-enrichment proves effective. Furthermore, they highlight that derived resource allocation attributes serve as novel diagnostic tools for predicting adaptive responses under global change scenarios.

## Supplementary Material

Supplementary_Material_tpag109(1)

## Data Availability

The original data are available from the corresponding author upon reasonable request.

## Notes

Frederick C. Meinzer holds the position of Editor for *Tree Physiology* and has not peer reviewed or made any editorial decisions for this paper.
