# Supplementary material for: Nutrient-mediated tree growth responses are governed by internal resource allocation: intra-organ water–carbon trade-offs and inter-organ carbon concentration gradient
Source: Tree Physiol. 2026 Aug 8;46(9):tpag109. doi: 10.1093/treephys/tpag109 (PMC13565153; doi:10.1093/treephys/tpag109)
Supplement: Supplementary file 1 [file supplementary_material_tpag109(1).docx]

**Supplementary material:**

**Nutrient-mediated tree growth responses are governed by internal resource allocation: intra-organ water-carbon trade-offs and inter-organ carbon concentration gradient**

Hui Shao^1,2^, Huimin Wang^1,2^, Frederick C. Meinzer^3^, Xiaoqin Dai^1,2^, Shengwang Meng^1,2^, Liang Kou^1,2^, Decai Gao^1,2^, Fusheng Chen^4^, Xiaoli Fu^1,2,4*^

^1^Qianyanzhou Ecological Research Station, Key Laboratory of Ecosystem Network Observation and Modeling, Institute of Geographic Sciences and Natural Resources Research, Chinese Academy of Sciences, 11A Datun Road, Chaoyang District, Beijing 100101, China

^2^College of Resources and Environment, University of Chinese Academy of Sciences, No. 1 Yanqihu East Road, Huairou District, Beijing 101408, China

^3^Department of Forest Ecosystems and Society, Oregon State University, 1500 SW Jefferson Way, Corvallis, Oregon 97331, USA

^4^Jiangxi Provincial Key Laboratory of Silviculture, College of Forestry, Jiangxi Agricultural University, No. 1101 Zhimin Road, Economic & Technological Development Zone, Nanchang, Jiangxi 330045, China

*Corresponding author: Xiaoli Fu

Institute of Geographic Sciences and Natural Resources Research, Chinese Academy of Sciences, Beijing 100101, China

Phone: 86-10-6488-9592

Fax: 86-10-6488-9592

Email: fuxl@igsnrr.ac.cn

Figure S1 (a) Differences in diameter at breast height (DBH) of *C. lanceolata* among treatments before nutrient addition (in 2012). (b) Effects of nutrient addition on DBH of *C. lanceolata* in 2023. Normality and homogeneity of variance are shown in Tables S1 and S2, respectively. Box plots show mean values (black squares) and median values (black solid lines). N, N addition; P, P addition; N+P, combined N+P addition. Different letters indicate significant differences between treatments (*P* < 0.05).

Figure S2 Density histograms of regression coefficients from 500 bootstrap resamples, illustrating correlations between intra-organ water-carbon trade-offs and growth rate. (a) *ln*(*RWC*_rank_/*ST*_rank_)_twig_; (b) *ln*(*RWC*_rank_/*SS*_rank_)_TR_; (c) *ln*(*RWC*_rank_/*SS*_rank_)_AR_. Blue lines show 95% confidence intervals, and the red line marks the mean coefficient.

Figure S3 Hierarchical partitioning quantifying the relative contributions of key observable resource concentrations to growth rate variation. R_adj_^2^ is the adjusted R^2^. SS, soluble sugar; ST, starch. TR, transport root.

Figure S4 Residual diagnostic plots for the GAM model. (a) Normal Q-Q plot; (b) residuals vs. fitted values; (c) histogram of residuals; (d) observed vs. fitted values. The Q-Q plot (a) followed an approximately straight line, with only minor deviations at both tails, indicating an acceptable fit. In plot (b), residuals were randomly distributed with no clear pattern, suggesting no systematic relationship with predictions. The residual histogram (c) was approximately centered near zero and roughly symmetric, suggesting approximate normality. Plot (d) showed observed values closely aligning with the y = x line, demonstrating a good model fit. Collectively, the diagnostic plots confirm that the GAM model achieved a good fit to the data.

Table S1 Results of the Kolmogorov-Smirnov test for resource attributes and diameter at breast height under each treatment.

| Variable | | Control | | N | | P | | N+P | |
| --- | --- | --- | --- | --- | --- | --- | --- | --- | --- |
|  |  | Statistic | *P* | Statistic | *P* | Statistic | *P* | Statistic | *P* |
| Observable resource concentrations | SS_leaf_ | 0.07 | 0.94 | 0.07 | 0.97 | 0.06 | 0.99 | 0.09 | 0.75 |
|  | SS_twig_ | 0.09 | 0.70 | 0.09 | 0.67 | 0.06 | 0.96 | 0.09 | 0.75 |
|  | SS_TR_ | 0.11 | 0.72 | 0.08 | 0.97 | 0.14 | 0.42 | 0.09 | 0.89 |
|  | SS_AR_ | 0.09 | 0.93 | 0.08 | 0.98 | 0.08 | 0.97 | 0.07 | 0.99 |
|  | ST_leaf_ | 0.15 | 0.15 | 0.11 | 0.45 | 0.15 | 0.15 | 0.11 | 0.45 |
|  | ST_twig_ | 0.10 | 0.51 | 0.10 | 0.60 | 0.21 | 0.01 | 0.11 | 0.53 |
|  | ST_TR_ | 0.14 | 0.46 | 0.13 | 0.53 | 0.18 | 0.25 | 0.16 | 0.28 |
|  | ST_AR_ | 0.13 | 0.49 | 0.14 | 0.42 | 0.11 | 0.83 | 0.09 | 0.90 |
| Organ hydration status | RWC_leaf_ | 0.10 | 0.56 | 0.12 | 0.45 | 0.08 | 0.85 | 0.11 | 0.57 |
|  | RWC_twig_ | 0.14 | 0.19 | 0.08 | 0.80 | 0.10 | 0.52 | 0.07 | 0.94 |
|  | RWC_TR_ | 0.10 | 0.90 | 0.09 | 0.93 | 0.09 | 0.93 | 0.06 | 1.00 |
|  | RWC_AR_ | 0.15 | 0.40 | 0.14 | 0.47 | 0.18 | 0.19 | 0.08 | 0.97 |
| Intra-organ water-carbon trade-offs | *ln*(*RWC*_rank_/*SS*_rank_)_leaf_ | 0.06 | 0.97 | 0.11 | 0.58 | 0.12 | 0.40 | 0.14 | 0.21 |
|  | *ln*(*RWC*_rank_/*SS*_rank_)_twig_ | 0.07 | 0.92 | 0.09 | 0.78 | 0.19 | 0.03 | 0.08 | 0.83 |
|  | *ln*(*RWC*_rank_/*SS*_rank_)_TR_ | 0.08 | 0.98 | 0.13 | 0.59 | 0.13 | 0.57 | 0.07 | 0.99 |
|  | *ln*(*RWC*_rank_/*SS*_rank_)_AR_ | 0.18 | 0.20 | 0.19 | 0.17 | 0.07 | 0.99 | 0.09 | 0.91 |
|  | *ln*(*RWC*_rank_/*ST*_rank_)_leaf_ | 0.09 | 0.71 | 0.08 | 0.81 | 0.12 | 0.42 | 0.08 | 0.79 |
|  | *ln*(*RWC*_rank_/*ST*_rank_)_twig_ | 0.08 | 0.82 | 0.15 | 0.13 | 0.14 | 0.21 | 0.10 | 0.67 |
|  | *ln*(*RWC*_rank_/*ST*_rank_)_TR_ | 0.17 | 0.26 | 0.11 | 0.71 | 0.09 | 0.92 | 0.06 | 1.00 |
|  | *ln*(*RWC*_rank_/*ST*_rank_)_AR_ | 0.13 | 0.63 | 0.19 | 0.16 | 0.13 | 0.61 | 0.10 | 0.81 |
| Inter-organ carbon concentration gradient | *SS_leat-twig_* | 0.06 | 0.96 | 0.09 | 0.82 | 0.09 | 0.64 | 0.09 | 0.69 |
|  | *SS_AR-TR_* | 0.13 | 0.53 | 0.10 | 0.88 | 0.09 | 0.95 | 0.09 | 0.93 |
|  | *ST_leat-twig_* | 0.06 | 0.99 | 0.11 | 0.58 | 0.16 | 0.09 | 0.11 | 0.42 |
|  | *ST_AR-TR_* | 0.11 | 0.74 | 0.06 | 1.00 | 0.08 | 0.97 | 0.10 | 0.88 |
| Diameter at breast height | DBH_2012_ | 0.10 | 0.56 | 0.09 | 0.77 | 0.08 | 0.90 | 0.06 | 0.99 |
|  | DBH_2023_ | 0.05 | 0.59 | 0.05 | 0.59 | 0.05 | 0.67 | 0.07 | 0.33 |

Note: SS, soluble sugar; ST, starch; RWC, relative water content. AR, absorptive root; TR, transport root. N, N addition; P, P addition; N+P, combined N+P addition. DBH_2012_, the diameter at breast height in 2012; DBH_2023_, the diameter at breast height in 2023. *P* > 0.05 indicates normality is satisfied. Only ST_twig_ and *ln*(*RWC*_rank_/*SS*_rank_)_twig_ under P addition fail to satisfy normality. Significance testing for non-normal data used Tamhane’s T2 test.

Table S2 Results of the homogeneity test for resource attributes and diameter at breast height across treatments.

| Variable | | Levene statistic | *P* | Variable | | Levene statistic | *P* |
| --- | --- | --- | --- | --- | --- | --- | --- |
| Observable resource concentrations | SS_leaf_ | 3.02 | 0.03 | Intra-organ water-carbon trade-offs | *ln*(*RWC*_rank_/*SS*_rank_)_leaf_ | 4.09 | 0.01 |
|  | SS_twig_ | 5.02 | 0.00 |  | *ln*(*RWC*_rank_/*SS*_rank_)_twig_ | 3.12 | 0.03 |
|  | SS_TR_ | **2.04** | **0.11** |  | *ln*(*RWC*_rank_/*SS*_rank_)_TR_ | **2.58** | **0.06** |
|  | SS_AR_ | **1.62** | **0.19** |  | *ln*(*RWC*_rank_/*SS*_rank_)_AR_ | **0.78** | **0.51** |
|  | ST_leaf_ | **0.41** | **0.74** |  | *ln*(*RWC*_rank_/*ST*_rank_)_leaf_ | **0.78** | **0.50** |
|  | ST_twig_ | 9.33 | 0.00 |  | *ln*(*RWC*_rank_/*ST*_rank_)_twig_ | **1.03** | **0.38** |
|  | ST_TR_ | **1.05** | **0.37** |  | *ln*(*RWC*_rank_/*ST*_rank_)_TR_ | 2.95 | 0.04 |
|  | ST_AR_ | 4.31 | 0.01 |  | *ln*(*RWC*_rank_/*ST*_rank_)_AR_ | 4.44 | 0.01 |
| Organ hydration status | RWC_leaf_ | **0.52** | **0.67** | Inter-organ carbon concentration gradient | *SS_leat-twig_* | 4.27 | 0.01 |
|  | RWC_twig_ | **1.67** | **0.17** |  | *SS_AR-TR_* | 3.10 | 0.03 |
|  | RWC_TR_ | **2.57** | **0.06** |  | *ST_leat-twig_* | **2.15** | **0.09** |
|  | RWC_AR_ | 5.42 | 0.00 |  | *ST_AR-TR_* | **2.02** | **0.11** |
| Diameter at breast height | DBH_2012_ | **2.16** | **0.09** |  |  |  |  |
|  | DBH_2023_ | **2.06** | **0.10** |  |  |  |  |

Note: SS, soluble sugar; ST, starch; RWC, relative water content. AR, absorptive root; TR, transport root. DBH_2012_, the diameter at breast height in 2012; DBH_2023_, the diameter at breast height in 2023. *P* > 0.05 indicates the data is homogeneous and is shown in bold. Significance testing for heteroscedastic data used Tamhane’s T2 test.

Table S3 Performance comparison of linear regression and quadratic polynomial regression for relationships between key resource attributes and growth rate.

| Attribute | | Linear regression | | | Quadratic polynomial regression | | |
| --- | --- | --- | --- | --- | --- | --- | --- |
|  |  | RMSE | MAE | R_adj_^2^ | RMSE | MAE | R_adj_^2^ |
| Observable resource concentrations | ST_twig_ | **0.3327** | **0.2780** | **0.1465** | 0.3436 | 0.2877 | 0.1263 |
|  | SS_TR_ | **0.3414** | **0.2851** | **0.1407** | 0.3441 | 0.2854 | 0.1226 |
| Intra-organ water-carbon trade-offs | *ln*(*RWC*ᵣₐₙₖ/*ST*ᵣₐₙₖ)_twig_ | 0.3150 | 0.3239 | 0.0992 | **0.3084** | **0.2887** | **0.1290** |
|  | *ln*(*RWC*ᵣₐₙₖ/*SS*ᵣₐₙₖ)_TR_ | **0.3491** | **0.2947** | **0.1023** | 0.3821 | 0.3141 | 0.0900 |
|  | *ln*(*RWC*ᵣₐₙₖ/*SS*ᵣₐₙₖ)_AR_ | **0.3474** | **0.2911** | **0.0784** | 0.3541 | 0.2970 | 0.0588 |
| Inter-organ carbon concentration gradient | *SS_AR-TR_* | **0.3491** | **0.2885** | **0.0701** | 0.3656 | 0.2967 | 0.0592 |

Note: Best statistical indicators between models are marked in bold. RMSE, root mean squared error; MAE, mean absolute error; R_adj_^2^ is the adjusted R^2^. SS, soluble sugar; ST, starch; RWC, relative water content. AR, absorptive root; TR, transport root.

Table S4 Results of linear and quadratic polynomial regression analyses showing non-significant relationships between tree growth rate and resource attributes.

| Observable resource concentrations/RWC | Linear regression | | Quadratic polynomial regression | | Intra-organ water-carbon trade-offs | Linear regression | | Quadratic polynomial regression | | Inter-organ carbon concentration gradient | Linear regression | | Quadratic polynomial regression | |
| --- | --- | --- | --- | --- | --- | --- | --- | --- | --- | --- | --- | --- | --- | --- |
|  | R_adj_^2^ | *P* | R_adj_^2^ | *P* |  | R_adj_^2^ | *P* | R_adj_^2^ | *P* |  | R_adj_^2^ | *P* | R_adj_^2^ | *P* |
| SS_leaf_ | -0.02 | 0.83 | 0.04 | 0.16 | *ln*(*RWC*ᵣₐₙₖ/*SS*ᵣₐₙₖ)_leaf_ | -0.02 | 0.77 | 0.00 | 0.38 | *SSₗₑₐ_f-twig_* | -0.01 | 0.42 | -0.03 | 0.66 |
| SS_twig_ | 0.02 | 0.16 | 0.05 | 0.13 | *ln*(*RWC*ᵣₐₙₖ/*SS*ᵣₐₙₖ)_twig_ | -0.01 | 0.41 | -0.02 | 0.56 | *STₗₑₐ_f-twig_* | -0.01 | 0.43 | -0.03 | 0.71 |
| SS_AR_ | 0.03 | 0.15 | 0.00 | 0.35 | *ln*(*RWC*ᵣₐₙₖ/*ST*ᵣₐₙₖ)_leaf_ | 0.00 | 0.32 | -0.02 | 0.61 | *ST_AR-TR_* | 0.00 | 0.28 | 0.00 | 0.38 |
| ST_leaf_ | 0.02 | 0.17 | 0.00 | 0.35 | *ln*(*RWC*ᵣₐₙₖ/*ST*ᵣₐₙₖ)_TR_ | 0.01 | 0.25 | -0.01 | 0.45 |  |  |  |  |  |
| ST_TR_ | 0.03 | 0.14 | 0.02 | 0.23 | *ln*(*RWC*ᵣₐₙₖ/*ST*ᵣₐₙₖ)_AR_ | 0.00 | 0.35 | 0.00 | 0.41 |  |  |  |  |  |
| ST_AR_ | -0.02 | 0.63 | -0.03 | 0.72 |  |  |  |  |  |  |  |  |  |  |
| RWC_leaf_ | -0.02 | 0.76 | -0.03 | 0.72 |  |  |  |  |  |  |  |  |  |  |
| RWC_twig_ | -0.02 | 0.85 | -0.05 | 0.98 |  |  |  |  |  |  |  |  |  |  |
| RWC_TR_ | -0.01 | 0.55 | 0.01 | 0.32 |  |  |  |  |  |  |  |  |  |  |
| RWC_AR_ | -0.01 | 0.42 | -0.02 | 0.52 |  |  |  |  |  |  |  |  |  |  |

Note: R_adj_^2^ is the adjusted R^2^. SS, soluble sugar; ST, starch; RWC, relative water content. AR, absorptive root; TR, transport root.
